# Elevated glucose promotes DNA replication and cancer cell growth through pRB-E2F1

**DOI:** 10.21203/rs.3.rs-3126261/v1

**Published:** 2023-07-10

**Authors:** Qi Zhou, Dongmei Bai, Jacob Schrier, Xuan Liu

**Affiliations:** Department of Biochemistry, University of California, Riverside, CA 92521

**Keywords:** E2F1, pRB, RRM2, high glucose, cancer

## Abstract

Although epidemiological studies have highlighted a link between hyperglycemia and increased risk of cancer, knowledge about the molecular mechanism behind the link is still limited. In this study, we report that high glucose levels (HG) enhance DNA replication, leading to tumor cell growth. Moreover, through genome-wide analyses, we identify E2F1 as the core transcription factor for this HG-induced cell adaptation. Inhibition of E2F1 abrogates the HG-induced DNA synthesis and cell growth, supporting the role of E2F1 in this process. Furthermore, we demonstrate that elevated glucose levels enhance pRB phosphorylation, which plays a role in E2F1 activation. Interestingly, among HG-induced E2F1 target genes, RRM2 (Ribonucleotide Reductase Regulatory Subunit M2) participates in the nucleotide synthesis by catalyzing the generation of the essential dNTP for DNA replication. We show that HG increases cellular dNTP levels in E2F1-RRM2-dependent manner, which correlates to enhanced DNA synthesis and cancer cell growth. Collectively, our findings decipher a pRB-E2F1-RRM2 dependent link between hyperglycemia and cancer cell proliferation and provide a molecular mechanism by which hyperglycemia directs tumor cells to DNA replication.

## Introduction

Cancer is characterized by uncontrolled cell proliferation with sustaining proliferative signaling as a classic hallmark (Hanahan, 2022; Hanahan & Weinberg, 2000, 2011). In many circumstances, transcription factors are pivotal effectors for altered proliferative signaling pathways and function to promote uncontrolled cell growth through regulation of transcription. Ultimately, altered signaling cascades lead to the process of DNA synthesis and cell cycle progression for excessive cell proliferation.

Diabetes mellitus is a group of metabolic disorders triggered by dysregulation of glucose metabolism resulting in hyperglycemia. It is categorized into two main types: type 1 (T1DM) and type 2 (T2DM), both have been linked to an increased risk of various cancers (Szablewski, 2014; Wang et al., 2020). Given that glucose is a key nutrient source for cell survival, especially for the rapidly proliferating tumor cells (Palm & Thompson, 2017), it is not surprising that elevated glucose level has been suggested as a leading risk factor for cancer in the past decade (Tran et al., 2022). In fact, hyperglycemia has been widely accepted as a major biological link between diabetes and cancer due to the Warburg effect: a higher dependency of tumor cells on glycolysis for continuously producing ATP energy molecule (Giovannucci et al., 2010b, 2010a; Szablewski, 2014; Warburg, 1956). Besides the direct effect on the cancer cells, elevated glucose levels can sustain an uncontrolled and everlasting chronic inflammatory state, creating a tumor favorable microenvironment to facilitate tumor development and metastasis (Chang & Yang, 2016). While hyperglycemia’s role in cancer has been studied, a comprehensive understanding of the molecular pathway by which hyperglycemia induces cancer growth remains lacking.

To systematically investigate this and to identify the role of key transcription factors in this process, we carried out high throughput RNA-seq analysis. Our data revealed that the core transcriptional regulator E2F1 plays a critical role in re-directing cancer cells to DNA synthesis and cell proliferation under elevated glucose conditions. Moreover, we showed that elevated glucose enhances pRB hyper-phosphorylation, leading to E2F1 activation. Interestingly, among HG-induced E2F1 target genes, RRM2 has been shown to participate in the nucleotide synthesis by generating essential dNTP for DNA replication. Our data suggested that HG leads to RRM2-dependent increase of cellular dNTP levels, which correlates to DNA synthesis and cancer cell growth. Together, our finding uncovers a new molecular mechanism for enhancing cell growth by elevated glucose and sheds light on the significance of the pRB/E2F axis as a potential therapeutic target in the tumor-bearing diabetic patients.

## Results

### Elevated glucose prompts DNA replication and cell growth

**Figure 1.**
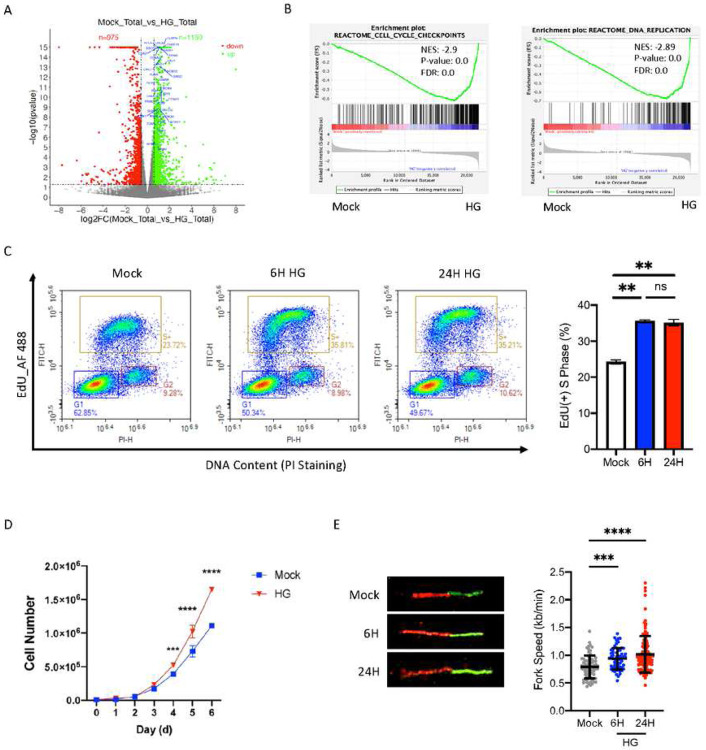
Elevated glucose prompts DNA replication and cell growth. A. Volcano plot depicting differential gene expression in HCT116 cells following HG exposure (Green: up, Red: down). Dashed lines denote the cutoffs for log2(FC) (± 0.58) and −log10(p value) (1.3). Triangles (Δ) represent data points exceeding the cap. The positions of DNA synthesis genes are labeled in blue. B. The top 2 significant enriched gene set of REACTOME pathways from GSEA analysis. C. Cell cycle profiles of EdU incorporation and DNA content in HCT116 treated with HG at the indicated time. The left panel is the representative analysis of flow cytometry. G1, S+ and G2 of the cell cycle were gated as indicated. The right panel indicates the percentage of EdU incorporated cells in the S phase. D. Cell growth curve of HCT116 cells under 5mM (Mock) and 25 mM (HG) conditions. E. DNA replication fork progression of control and HG-treated HCT116 cells was determined by DNA fiber assay at the indicated time. The right panel summarizes measuring of 100–150 spread DNA fibers in HCT116 cells for each condition. Results are displayed in mean ± SD for n=3 replicates.

To understand the molecular mechanism for HG-induced cell proliferation, we performed RNA-seq analysis on HG treated colon cancer HCT116 cells. Differential expression analysis revealed 1705 up regulated and 1316 down regulated mRNA transcripts (padj<0.05) in response to HG treatment (Fig. 1A). Furthermore, gene set enrichment analysis (GSEA) (Subramanian et al., 2005) revealed that DNA replication and cell cycle checkpoints were the top two REACTOME pathways that are strongly enriched following HG treatment (Fig. 1B). Consistently, Gene Ontology (GO) analysis of all up-regulated genes in HG-treated cells also revealed significant enrichment in the DNA replication promoting genes (Fig. S1A). Those identified genes are illustrated on volcano plot (Fig. 1A) and listed in Table 1. Interestingly, GO analysis of all down regulated genes revealed cellular response to glucose starvation (Fig. S1B). Based on the gene expression profile analyses, we hypothesized that, upon HG treatment, cancer cells are directed to DNA replication for cell proliferation potentially through transcription activation.

To test our hypothesis, we first investigate the effect of HG on DNA replication by measuring the percentage of cells undergoing DNA synthesis using 5-ethynyl-2′-deoxyuridine (EdU) incorporation assay. The results show, compared to 5mM glucose control, an increased percentage of HCT116 cells in S phase at both 6 h and 24 h following HG treatment (Fig. 1C). Moreover, cell proliferation assays revealed that cells treated with HG displayed a growth advantage compared with the cells in control conditions (Fig. 1D). To seek for the direct evidence for enhanced DNA replication in HG-treated cells, we determined replication fork speed of a progressing fork in the cell using the DNA fiber assay. The results suggest an increase of the fork speed in an undergoing replication fork upon HG treatment (Fig. 1E). Measuring of 100–150 spread DNA fibers for each condition further confirmed the results (Fig. 1E). To exclude the possibility that HG-induced phenotype is specifically observed in HCT116 cells, we tested the HG response in another cell line H460 derived from lung cancer. The results show similar HG enhancing effects on DNA replication and cell proliferation as compared with those observed in HCT116 cells (Fig. S1C-E). Together, these results confirmed our hypothesis that elevated glucose levels re-direct cells to DNA synthesis and cell proliferation.

### E2F1 plays a crucial role in high glucose-induced DNA replication and cell growth

**Figure 2.**
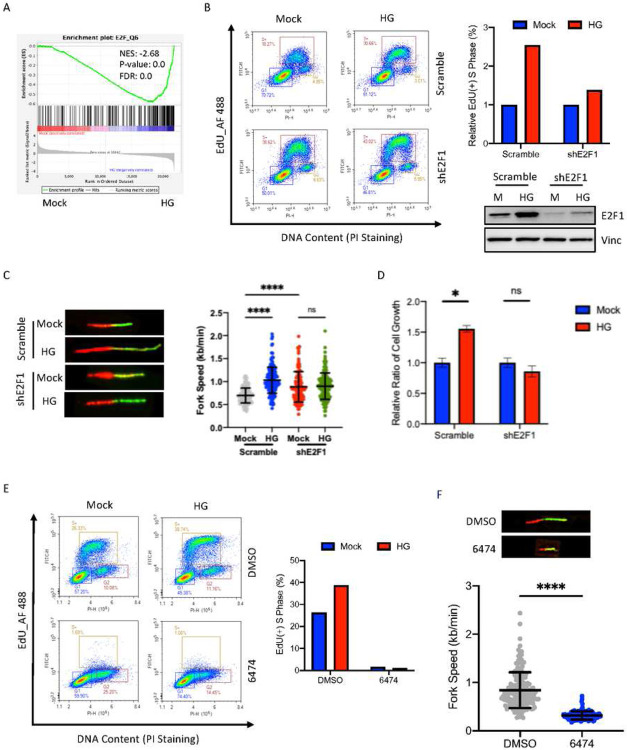
E2F1 plays a crucial role in high glucose-induced DNA replication and cell growth. A. The most significant enriched transcription factor revealed by GSEA. B. Cell cycle profiles of EdU incorporation and DNA content in HG-treated H460 cells in the presence of scramble control or shE2F1. The right panel indicates the relative percentage of EdU incorporated cells in the S phase following HG exposure, with or without E2F1 knock down. The E2F1 levels were verified by Western analysis. C. DNA replication progression of HG-treated H460 cells with or without E2F1 knock down. D. Cell growth assay of control or E2F1 knockdown H460 cells following HG. E. HCT116 cells were treated with DMSO or the E2F inhibitor HLM006474. EdU incorporation and DNA staining were analyzed by flow cytometry following HG exposure. The right panel indicates the percentage of EdU-positive cells in the S phase. F. DNA replication fork progression of HCT116 cells treated with HLM006474 was determined by DNA fiber assay following HG exposure. The bottom panel summarizes measuring of 100–150 spread DNA fibers in H460 cells for each condition. Results are displayed in mean ± SD for n=3 replicates.

Next, we performed GSEA analysis to identify key transcription factors responsible for this HG-induced cell adaptation. Our analyses revealed E2F1 as the top transcription factor (Fig. 2A, Table 2). To confirm the role of E2F1 in HG-induced DNA synthesis and cell proliferation, we employed lentivirus-mediated RNAi approach to knockdown E2F1 (Fig. 2B). Our results show that the HG-induced cells entering into S phase were significantly reduced upon E2F1 inhibition (Fig. 2B). Similarly, HG-induced increases in DNA replication fork speed and cell growth were also reduced in E2F1 knockdown cells (Fig. 2C, 2D).

We note that, compared to controls, E2F1 knocked down alone led to more EdU incorporated cells in the S phase (Fig. 2B) as well as increased DNA replication fork speed (Fig. 2C). Since the activator E2F family members (E2F1–3) have been shown to have extensive functional redundancy and overlap, we speculate that other E2F family members may offset the E2F1 knocked down effect, resulting in increased cells in the S phase and DNA synthesis. To test this possibility, we employed a pan-E2F inhibitor HLM006474 that blocks chromatin accessibility of E2F family members in cells (Y. Ma et al., 2008). Strikingly, HLM006474 completely abolished HCT116 cells entering into S phase in the presence and absence of HG (Fig. 2E). Similarly, a remarkable DNA replication fork arrest was also detected in HLM006474-treated cells (Fig. 2F). To exclude the potential cell type specific effect, we knock-downed E2F1 in HCT116 cells (Fig. S2A) and inhibited E2F1 with the inhibitor in H460 (Fig. S2B) and obtained similar HG effect on DNA replication and cell proliferation. Together, these results indicate that E2F1 plays a crucial role in promoting DNA replication and cell proliferation following HG treatment.

### Elevated glucose induces E2F1-dependent transactivation through pRB phosphorylation

**Figure 3.**
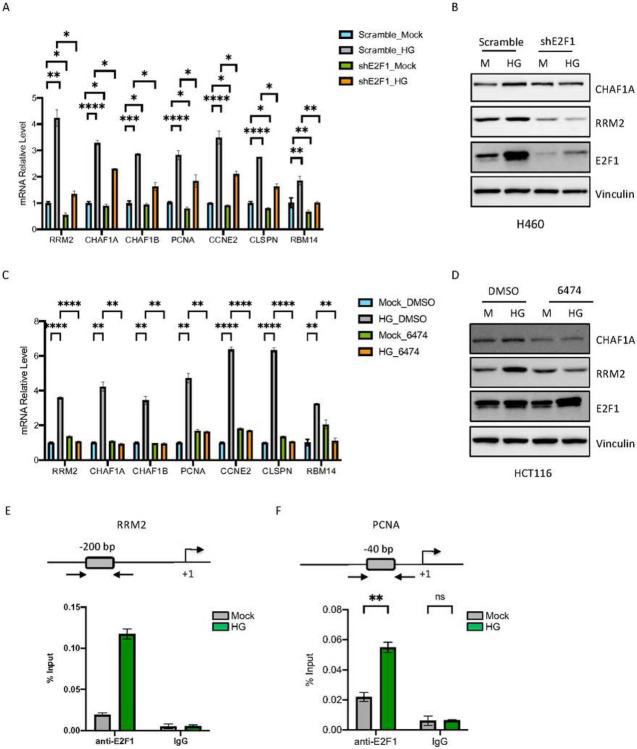
E2F1 regulates transcription of DNA replication genes in response to elevated glucose. A. RT-qPCR of top DNA replication genes in control (scramble) or E2F1 knock down H460 cells following HG exposure. B. Western analysis of CHAF1A, RRM2 and E2F1 protein levels from the same treatment as indicated in 3A. C. RT-qPCR of top DNA replication genes in control or HLM006474 treated HCT116. D. Western blot analysis for CHAF1A, RRM2 and E2F1 from the same treatment as indicated in 3C. E and F. Top: schematic representation of the RRM2 (3E) or PCNA (3F) promoters. ChIP-qPCR analysis of E2F1 binding to the RRM2 and PCNA promoters in the HCT116 cells following HG exposure. Results are displayed in mean ± SD for n=3 replicates.

To further establish the role of E2F1 in HG-induced DNA synthesis and cell growth, we confirmed its effect on up-regulation of 7 identified DNA replication genes (Table 1, Fig. 1A) by RT-PCR. As shown in Figure 3A, compared to controls, HG significantly increased the mRNA levels of RRM2, CHAF1A, CHAF1B, PCNA, CCNE2, CLSPN and RBM14. E2F1 knockdown, however, significantly reduced the HG-induced up-regulation. Importantly, E2F1 knockdown also reduced HG-induced RRM2 and CHAF1A protein levels (Fig. 3B), suggesting functional relevance of the regulation. Furthermore, we showed that treating cells with the E2F1 inhibitor HLM006474 also blocks HG-induced mRNA and protein levels of DNA replication-associated genes (Fig. 3C and 3D). Consistently, overexpression of E2F1 up-regulated the mRNA and protein levels of the DNA replication genes and reduced HG-induced up-regulation in cells (Fig. S3A and S3B). These results suggest that E2F functions as a transcription factor up-regulating DNA replication genes following HG treatment.

Several groups have reported that E2F1 binds to the RRM2 promoter and activates its transcription (Fang et al., 2015; Mazzu et al., 2018; Rasmussen et al., 2016). We thus carried out ChIP analysis to investigate whether elevated glucose affects the ability of E2F1 to bind to DNA. The assay confirmed the binding of E2F1 to the RRM2 promoter (Fig. 3E) as well as to the PCNA promoter (Fig. 3F). Importantly, exposure of cells to HG significantly enhanced the binding of E2F1 to both promoters. Together, these results suggest that elevated glucose up-regulates DNA replication genes through E2F1-dependent transactivation.

**Figure 4.**
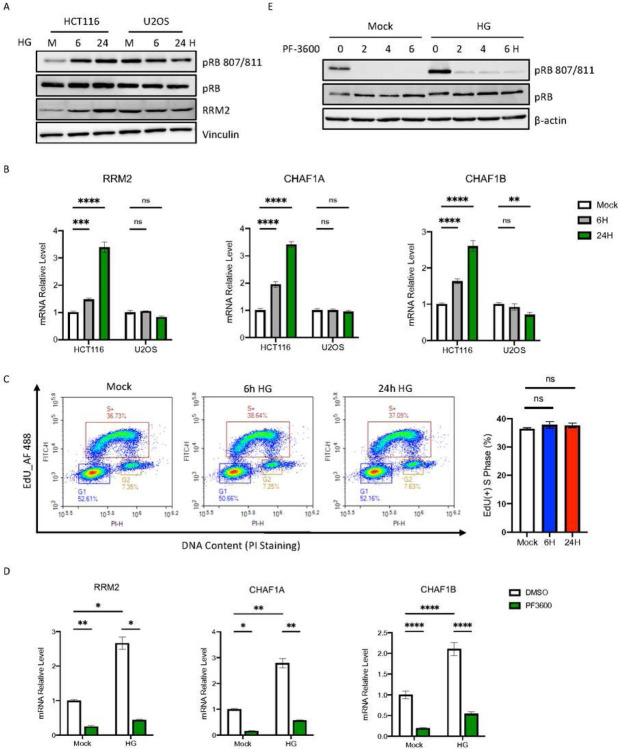
HG-induced pRB phosphorylation is required for activation of DNA replication genes. A. Western blot analysis of total pRB, Ser807/Ser811-phosphorylated pRB and RRM2 in HCT116 and U2OS cells following HG exposure. B. RT-qPCR of top DNA replication genes in HCT116 and U2OS cells following HG treatment. C. Cell cycle profiles of EdU incorporation and DNA content in U2OS cells following HG treatment at the indicated time. The right panel indicates the percentage of EdU-labeled cells in the S phase. D. HCT116 cells were treated with the CDK inhibitor PF-3600 and RT-qPCR of top DNA replication genes was performed following HG exposure. E. Western blot analysis of total pRB and Ser807/Ser811-phosphorylated pRB from the same treatment as indicated in 4D. Results are displayed in mean ± SD for n=3 replicates.

Interestingly, a previous study has suggested that treating cells with HG leads to pRB hyper-phosphorylation (Annicotte et al., 2009). pRB is the target of phosphorylation by CDK2/4/6 in the G1 phase of the cell cycle. Generally, once hyper-phosphorylated, pRB alleviates repression of E2F, leading to activation of E2F1-dependent transcription. To test the role of pRB phosphorylation in HG-induced E2F transactivation, we treated cells with HG and assay pRB phosphorylation using phosphorSer807/811-specific antibody. As shown in Figure 4A, increased pRB phosphorylation was clearly observed following HG treatment in HCT116 cells, suggesting pRB hyperphosphorylation potentially contributed to E2F1 activation. Interestingly, perhaps due to higher levels of pRB phosphorylation in the cell, treating U2OS cells with HG didn’t further enhance pRB phosphorylation (Fig. 4A). Significantly, compared to HG-treated HCT116 cells, treating U2OS cells with HG also failed to up-regulate DNA replication genes (Fig. 4B and S4A) and to enhance DNA synthesis (Fig. 4C). To confirm these results, we treated HCT116 cells with the CDK2/4/6 inhibitor PF-3600 (Freeman-Cook et al., 2021) and showed inhibition of pRB phosphorylation (Fig. 4E) also blocks HG-induced up-regulation of DNA replication genes (Fig. 4D and S4B). These results suggest that elevated glucose up-regulates DNA replication associated genes through regulating the pRB-E2F pathway.

### Regulation of intracellular dNTP levels by HG is dependent on the E2F1-RRM2 axis

**Figure 5.**
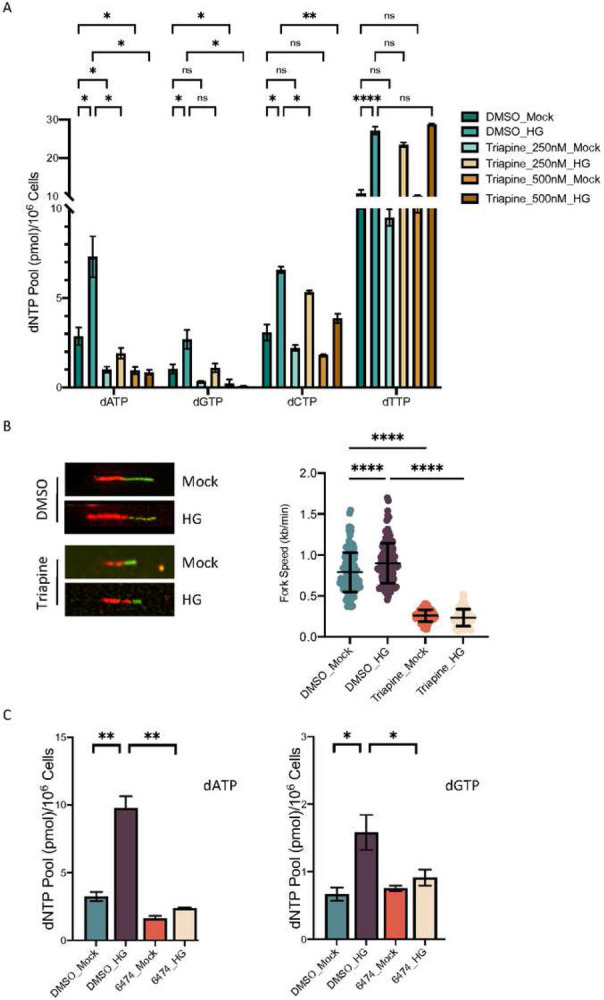
Regulation of intracellular dNTP levels by HG is dependent on the E2F1-RRM2 axis. A. Intracellular dNTP levels in HCT116 cells treated with or without Triapine and HG as indicated. B. DNA replication fork progression of Triapine-treated HCT116 cells following HG exposure. The right panel summarizes measuring of 100–150 spread DNA fibers for each condition. C. HCT116 cells were treated with the E2F inhibitor HLM006474. Intracellular dATP and dGTP levels were determined following HG. Results are displayed in mean ± SD of three separate experiments.

The role of RRM2 as a proto-oncogene has been recently recognized. RRM2 is an essential component in the holoenzyme ribonucleotide reductase (RNR) that is important for reducing the 2’ carbon of NDP to produce dNDP, a rate-limiting step in the DNA *de novo* pathway. Our finding that HG up-regulates both RRM2 mRNA and protein levels prompts us to test its role in regulating intracellular dNTP levels. Using a previously described PCR-based method (Purhonen et al., 2020) the intracellular dATP, dGTP, dCTP and dTTP levels were measured and calculated at 2.86, 1.04, 3.07, and 10.82 pmol/10^6^ cells, respectively. Treating cells with HG led to a rapid and robust increase of all four dNTP levels in the cells (Fig. 5A). To establish the role of RNR in the HG-induced upregulation of dNTP, we treated cells with the RNR inhibitor Triapine at two concentrations, 250 or 500 nM, and observed reduced intracellular dATP and dGTP levels under both conditions. The Triapine treatment also reduced dCTP and dTTP, but to a lesser extent, which is consistent with a previous report (Lin et al., 2011). Importantly, inhibition of RNR clearly prevented HG-induced DNA replication fork progression (Fig. 5B). Finally, we verified the role of E2F1 in HG-induced dNTP up-regulation. As shown in Figure 5C, treating cells with the E2F inhibitor 6476 clearly blocks dATP and dGTP up-regulation following HG treatment. Together, these results suggest that elevated glucose enhances dNTP levels through activation of the E2F1-RRM2 axis.

### Inhibition of E2F1-RRM2 axis alleviates high glucose-induced cancer cell growth

**Figure 6.**
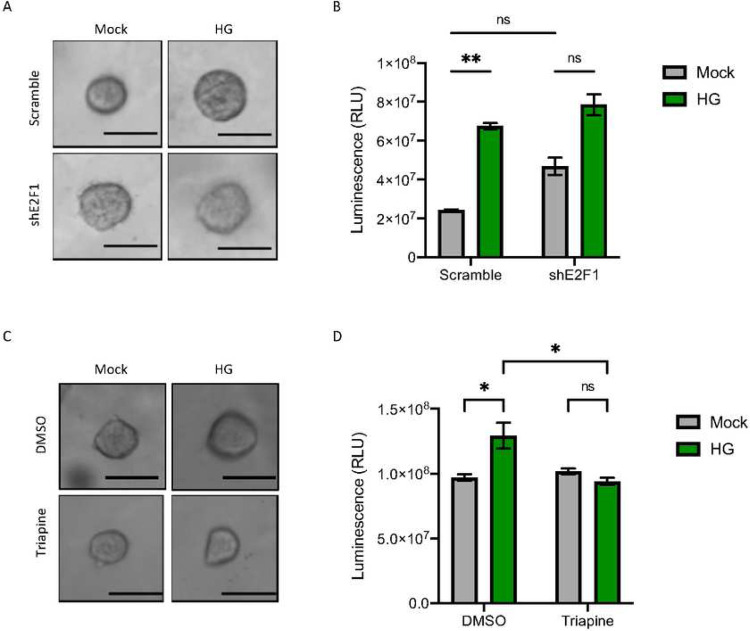
Inhibition of the E2F1-RRM2 axis alleviates HG-induced cancer cell growth. A. Representative bright field images of H460 derived spheroids in the presence of scramble control or shE2F1 for 6 days following HG exposure. Scale bar: 100μm. B. ATP luminescence signals represented for the total spheroid’s viability on the plate. C. Representative bright field images of HCT116-derived spheroids in the presence or absence of Triapine for 6 days following HG exposure. D. ATP luminescence signals represented for the total spheroid’s viability on the plate. Results are displayed in mean ± SD of three separate experiments.

We next investigated the contribution of the E2F1-RRM2 axis to cancer cell growth following HG treatment. To better mimic the *in vivo* environment, we established a short-term three dimensional (3D) tumor spheroid culture (Zoetemelk et al., 2019). As shown in Figure 6A, H460 cells formed spheroid-like round or spherical structures in the 3D cultures. Upon HG treatment, increased size of the spheroids was observed, indicating a stronger cell growth compared with non-HG treated spheroids (Fig. 6A). To confirm the cell growth potential, overall cell viability of total spheroids in the 3D cultures was further determined following HG treatment (Fig. 6B). These results provide additional support for the finding that elevated glucose re-directs cells to cell growth.

To investigate the role of E2F1 in HG-induced spheroid growth, we employed lentivirus-mediated RNAi approach to knockdown E2F1. The results show that compared with scramble controls, HG-induced spheroid growth was reduced upon E2F1 inhibition (Fig. 6A). Importantly, HG-induced cell viability of total spheroids in the 3D cultures was also reduced in E2F1 knockdown cells (Fig. 6B).

Next, we investigated the role of RRM2 in HG-induced spheroid growth by treating cells with the RNR inhibitor Triapine. As shown in Figure 6C, HG-induced spheroid growth was indeed blocked upon the RNR inhibition. As expected, HG-induced cell viability of total spheroids in the 3D cultures was also blocked by the inhibition (Fig. 6D). Taken together, these results demonstrate elevated glucose promotes cancer cell growth through E2F1-RRM2 activation.

## Discussion

Diabetes mellitus and cancer have a tremendous effect on human health worldwide. Although a number of epidemiological studies have highlighted the link between two diseases (Giovannucci et al., 2010b; Gordon-Dseagu et al., 2013; Wang et al., 2020), the molecular mechanism by which hyperglycemia promotes cancer cell growth remains well defined. In this study, we identified E2F1 as the core transcriptional regulator involved in re-directing cells to DNA replication and cell proliferation under elevated glucose conditions. Among HG-induced E2F1 target genes, we show that activation of RRM2 leads to up-regulation of intracellular dNTP levels, which plays a role in DNA synthesis and cancer cell growth. Together, our findings provide a molecular mechanism by which hyperglycemia promotes cancer cell proliferation.

E2F transcription factors are downstream effectors of the tumor suppressor pRB and have a pivotal role in controlling cell-cycle progression. E2Fs also participate in cellular processes beyond the cell cycle, including apoptosis, differentiation and development. However, the role of E2Fs in re-directing cancer cells to proliferation following HG has not been well investigated. By examining the transcriptome data of the HG-treated cells, DNA replication emerges as a significant signature. Furthermore, GSEA analysis revealed E2F1 as the core transcription factor, suggesting hyperglycemia potentially re-directs cancer cells into DNA replication through E2F1-mediated transcription. Interestingly, consistent with this notion, regulation of DNA replication genes has been reported in fin tissues in the diabetic zebrafish model (Leontovich et al., 2016). Furthermore, inhibition of GLUT1, a key rate-limiting factor for glucose uptake, blocked growth of pRB-positive triple negative breast cancer (TNBC) (Wu et al., 2020). Notably, pathway enrichment analysis of gene expression data in TNBC also suggests that the functionality of the E2F pathway contributed to the process. Together, those results imply a HG-regulated pRB-E2F1 axis in cancer cells. Clearly, it will be intriguing to further assess its contribution in pRB-positive cancer patients.

Emerging evidence has indicated that RRM2, the small subunit of RNR, is an important proto-oncogene and cancer therapeutic target (Aye et al., 2015). The importance of RNR for DNA replication relates to its central role in regulating dNTP levels. RRM2 expression is elevated in several cancer types and the level of the expression is highly correlated with tumor grades (Aird et al., 2014; Aye et al., 2015; X.-J. Ma et al., 2003). Furthermore, overexpression of RRM2 is often associated with chemo-resistant cancers. Interestingly, RRM2 has been shown a direct transcriptional target of several transcription factors including E2F1 (Fang et al., 2015; Rasmussen et al., 2016). Herein, our result that RRM2 can be transcriptionally activated by E2F1 following HG provides a new link between diabetes and cancer and potentially suggests a new avenue for targeted cancer therapy for diabetes patients. In fact, the RNR inhibitor Triapine employed in our study is currently undergoing clinical trials. It has been shown to increase sensitivity of chemotherapy and radiation therapy in cervical cancer cells (Kunos et al., 2010, 2013). Our data that Triapine treatment inhibits HG-induced DNA replication fork further validated its application in targeted cancer therapy for diabetes patients. Together, our data suggest a model of how cancer cells exert adaptation to hyperglycemia and illustrate the molecular mechanism of E2F to be the core transcription factor in activating the transcription of the downstream DNA replication genes, therefore promoting the DNA replication and cellular proliferation.

## Materials and Methods

### Cell culture and reagents

HCT116 cells were cultured in DMEM supplemented with 10% FBS. H460 cells were cultured in RPMI 1640 supplemented with 10% FBS. U2OS cells were cultured in McCoys 5A media supplemented with 10% FBS. HEK293T cells were cultured in DMEM supplemented with 10% heat inactivated FBS. For HG treatments, cells were exposed to 25mM glucose (Boston BioProducts) for 6 h or as indicated. To inhibit E2F activity, cells were treated with 40 μM pan-E2F inhibitor HLM006474 (Sigma-Aldrich) for 9 h (Y. Ma et al., 2008). To inhibit RRM2 activity, HCT116 cells were treated with 250 or 500 nM Triapine (Selleck Chemicals) as described (Lin et al., 2011). To inhibit pRB S807/811 phosphorylation, HCT116 cells were treated with 1μM PF-3600 (PF-06873600, Cayman Chemical) as indicated (Freeman-Cook et al., 2021).

The following antibodies were used for IB: anti-Vinculin (V9131, Sigma-Aldrich), anti-RRM2 (sc-398294, Santa Cruz), anti-E2F1 (sc-251, Santa Cruz), anti-CHAF1A (sc-133105, Santa Cruz), anti-Rb1 (#9309, Cell Signaling Technology), anti-phospho Rb1 (S807/811) (#8516, Cell Signaling Technology) and anti-b-actin (A3854, Millipore Sigma).

### RNA-seq and data analysis

Total RNA was isolated and purified from HCT116 cells in biological triplicates using Direct-zol RNA Miniprep Plus Kit (Zymo Research) according to the supplier’s instruction. RNA-seq libraries were then prepared following the manufacturer’s protocol of NEBNext Ultra II Directional RNA Library Prep Kit for Illumina (New England BioLabs). The sequencing was performed on Illumina NextSeq using single end 75bp read length. Sequencing reads were mapped to the human reference genome (GENCODE v34) using Salmon (Patro et al., 2017), followed by differential expression analysis using DESeq2 (Love et al., 2014). Gene set enrichment analysis (GSEA) (v4.2) was used to analyze the enrichment of REACTOME pathways and transcription factors among differentially expressed genes (Subramanian et al., 2005). Volcano plot was generated using ggplot2 (v3.4.0). GeneOntology biological process analysis was performed in ShinyGO (v0.75) (Ge et al., 2020).

### shRNA-mediated knock down

E2F1-targeting and scramble shRNA (Table 3) were cloned into pLKO.1 vector with puromycin selection marker. Lentivirus were produced by transfection of HEK 293T cells with the transfer plasmid and lentiviral packaging and VSVG plasmids. The virus was harvested 48 h post-transfection. H460 cells were transduced with the lentivirus in the presence of 10ug/ml polybrene (Millipore). After 48 h, transduced cells were selected with 1 μg/ml puromycin for 7 days.

### Cell cycle analysis

Cell cycle analysis was performed using the Click-iT Plus Edu Kit (#C10632, Thermo Scientific) according to the manufacturer’s protocol. In brief, 2 million cells post-treatment were harvested and fixed. EdU was then labeled with Alexa Fluor 488 picolyl azide for 30 min at room temperature. After labeling, total DNA content was stained with 20ng/ml PI (#P3566, Invitrogen) for 1 h at room temperature. Samples were then analyzed by flow cytometry on the NovoCyte platform (ACEA).

### DNA fiber assay

Cells were pulse-labeled with 25 μM CIdU (Sigma-Aldrich) for 20 min, followed by a second pulse of 250 μM IdU (Fisher Scientific) for another 20 min. Cells were harvested, lysed and DNA spread on slides as previously described (Halliwell et al., 2020). DNA fibers were further denatured in 2.5M HCl and blocked with 1% BSA in PBS with 0.1% Tween-20 (PBST) to reduce background. The labeled CIdU and IdU fibers were immunoblotted with the following primary antibodies (1:500 dilution) for 1 h at room temperature: rat monoclonal anti-BrdU antibody [BU1/75 (ICR1)] (#ab6326, Abcam) and mouse monoclonal anti-BrdU antibody (clone B44) (#BDB347580, Fisher Scientific). Secondary antibodies of Alexa Fluor 555 goat anti-rat IgG (#A21434, Thermo Scientific) and Alexa Fluor 488 F (ab′)2 goat anti−mouse IgG (#A-11017, Thermo Scientific) were used to at 1:500 dilution for 2 h at room temperature. Images of well spread DNA fibers were taken using Leica microscope with X 40 oil immersion objective. 100–150 well spread DNA fibers were collected for each condition. Double-labeled DNA fiber lengths were measured in Image J (v1.53k). The rate for DNA replication fork was estimated using the conversion of 2.59 kb/μm as described (Jackson & Pombo, 1998).

### RNA isolation and RT-qPCR

RNA was extracted with the TRIzol reagent (#15596018, Invitrogen) and then reverse-transcribed with the reverse transcription supermix (#1708841, Bio-Rad) according to the manufactures’ protocols. qPCR was performed using the SYBR supermix (#1708882, Bio-Rad) in the Bio-Rad CFX cycler with CFX Maestro software. Primers for qPCR are listed in Table 3. Results of mRNA relative levels were calculated by 2^−ΔΔCt^ in normalization to the GAPDH and relative to the control samples. All PCR reactions were performed in technical triplicates.

### ChIP-qPCR

Cells were cross-linked in 1% formaldehyde (#F1635, Sigma-Aldrich) for 10 min at room temperature and followed by attenuation of 125 mM glycine. Nuclei were isolated in NP-40 cell lysis buffer (50 mM Tris pH 8, 150 mM NaCl, 1% NP40, 15 mM EDTA) for 20 min on ice and spun down 5000 rpm for 5 min at 4°C. Nuclei were then lysed in nuclear lysis buffer (50 mM Tris pH 8, 150 mM NaCl, 10 mM EDTA, 1% SDS) for 10 min on ice. Lysed nuclei were then subjected to sonication to generate ~300bp chromatin fragments by confirmation on agarose gel. Immunoprecipitation was continued by incubating the sheared chromatin with E2F1 antibody (#3742, Cell Signaling) and IgG control (#2729, Cell Signaling) overnight at 4°C. Protein G beads (#10003D, Thermo Scientific) were added the following day and incubated on a rotator for 4h at 4°C. Immunoprecipitated beads were then washed twice in low salt wash buffer (0.1% SDS, 1% Triton X-100, 2 mM EDTA, 20 mM Tris pH 8, 150 mM NaCl), high salt wash buffer (0.1% SDS, 1% Triton X-100, 2 mM EDTA, 20 mM Tris pH 8, 500 mM NaCl), LiCl wash buffer (0.25 M LiCl, 1% NP40, 1% deoxycholate, 1 mM EDTA, 10 mM Tris pH 8) and once in TE buffer (1 mM EDTA, 10 mM Tris pH 8) and finally eluted in elution buffer (1% SDS, 0.1 M NaHCO3). The eluted immunoprecipitations and previously saved input samples were reverse cross-linked in a 65°C water bath overnight. Reverse cross-linked DNA was isolated by PCR purification kit (Qiangen).

ChIP-qPCR was performed using the SYBR supermix (Bio-Rad) in the Bio-Rad CFX cycler with CFX Maestro software. Primers for ChIP-qPCR are listed in Table 3. 1% of starting chromatin was used as input and technical triplicates were performed. The ChIP-qPCR data was analyzed with the Percent Input Method including normalization for both IgG levels and input chromatin going into the ChIP.

### Three-dimensional (3D) cultures

Cells were seeded at 2000 cells per well in a 96-well U-bottom plate (#353077, Corning). The 3D cell culture media was a mixture of the media for 2D cell culture supplemented with 10% Matrix (#A1413201, Thermo Scientific). On day 6, sphere colonies were firstly observed under microscope and images were taken using Leica microscope with X 5 bright field objective. The length and width for each spheroid were measured afterwards in Image J (v1.53k). The viability for spheroids was determined by CellTiter-Glo^®^ 3D Cell Viability Assay Kit (#G9681, Promega). Briefly, 100 μl CellTiter-Glo^®^ 3D Reagent was added into the wells to be determined, followed by shaking for 5 min. After incubation at room temperature for 25 min, the plate was read on the luminometer plate reader (Promega). The luminescence signals were measured and collected for evaluating the 3D cell growth viability.

### Intracellular dNTPs measurement

Intracellular dNTP levels were determined as previously described (Purhonen et al., 2020). Briefly, cell pellets were resuspended in 60% methanol (Fisher Scientific) and then incubated at 95°C for 3 min. The supernatant was collected after centrifugation and transferred into the Amicon Ultra-0.5ml centrifugal filter (#UFC500396, Millipore) for centrifugation again. After centrifugation, the flow through was saved and dried using Speed-Vac. The dried pellet was dissolved in 300 μl of sterile water and stored at −80°C. Determination of dNTP levels were performed by following the PCR based assay as previously described (Purhonen et al., 2020).

### Statistical analysis

All the data are represented as mean ± SD. All the statistical tests were done using Graphpad Prism 9. P values were also generated in Graphpad Prism 9, with *P <0.05, **P <0.01, ***P <0.001, ****P <0.0001, ns represents non-significance.
